# Transcription of *Tnfaip3* Is Regulated by NF-κB and p38 via C/EBPβ in Activated Macrophages

**DOI:** 10.1371/journal.pone.0073153

**Published:** 2013-09-02

**Authors:** Ting-Yu Lai, Shang-Duen Wu, Mong-Hsun Tsai, Eric Y. Chuang, Li-Ling Chuang, Li-Chung Hsu, Liang-Chuan Lai

**Affiliations:** 1 Institute of Molecular Medicine, National Taiwan University, Taipei, Taiwan; 2 Graduate Institute of Biomedical Electronics and Bioinformatics, National Taiwan University, Taipei, Taiwan; 3 Institute of Biotechnology, National Taiwan University, Taipei, Taiwan; 4 Bioinformatics and Biostatistics Core, Center of Genomic Medicine, National Taiwan University, Taipei, Taiwan; 5 YongLin Biomedical Engineering Center, National Taiwan University, Taipei, Taiwan; 6 Graduate Institute of Physiology, National Taiwan University, Taipei, Taiwan; 7 Department of Physical Therapy and Graduate Institute of Rehabilitation Science, Chang Gung University, Taoyuan, Taiwan; Centro Cardiologico Monzino IRCCS, Italy

## Abstract

Macrophages play a pivotal role in the immune system through recognition and elimination of microbial pathogens. Toll-like receptors (TLRs) on macrophages interact with microbial substances and initiate signal transduction through intracellular adapters. TLR4, which recognizes the lipopolysaccharides (LPS) on Gram-positive and Gram-negative bacteria, triggers downstream signaling mediators and eventually activates IκB kinase (IKK) complex and mitogen-activated protein kinases (MAPKs) such as p38. Previous reports revealed that, in addition to NF-κB, a core transcription factor of the innate immune response, the induction of some LPS-induced genes in macrophages required another transcription factor whose activity depends on p38. However, these additional transcription factors remain to be identified. In order to identify p38-activated transcription factors that cooperate with NF-κB in response to LPS stimulation, microarrays were used to identify genes regulated by both NF-κB and p38 using wild-type, IKK-depleted, and p38 inhibitor-treated mouse bone marrow-derived macrophages (BMDMs). *In silico* analysis of transcription factor binding sites was used to predict the potential synergistic transcription factors from the co-expressed genes. Among these genes, NF-κB and C/EBPβ, a p38 downstream transcription factor, were predicted to co-regulate genes in LPS-stimulated BMDMs. Based on the subsequent results of a chromatin immunoprecipitation assay and TNFAIP3 expression in C/EBPβ-ablated macrophages, we demonstrated that *Tnfaip3* is regulated by both NF-κB and p38-dependent C/EBPβ. These results identify a novel regulatory mechanism in TLR4-mediated innate immunity.

## Introduction

The innate immune system is the initial host response to microbes; it prevents infection and repairs tissues [1], [2]. During microbial infections, pathogen-associated molecular patterns derived from microbes, such as lipopolysaccharides (LPS), are recognized by pattern recognition receptors (PRRs), which then mount a defensive response [2]–[4]. In addition, endogenous molecules, called danger- (or damage-) associated molecular patterns, released by injured cells stimulate PRRs to initiate so-called “sterile inflammation,” which is crucial for tissue and wound repair [2], [3], [5]. In contrast to the beneficial effects of the innate immune response, dysregulation of pro-inflammatory cytokines has been linked to the pathogenesis of chronic inflammatory and infectious disease [3], [6], [7]. Thus, much research effort has been focused on understanding the regulation of innate immune responses, yet the underlying mechanisms remain imprecisely characterized.

Toll-like receptors (TLRs) are the best characterized PRRs and play an important role in the innate immune system [1], [2]. TLRs are transmembrane proteins composed of N-terminal leucine-rich repeats, a transmembrane region, and a cytoplasmic Toll/IL-1R homology (TIR) domain at the C-terminus. Among TLRs, TLR4 plays a central role in the recognition of both Gram-negative and Gram-positive bacteria [3], [8], [9]. TLR4 is the only TLR which can recruit four different adaptor proteins – myeloid differentiation primary response protein 88 (MyD88), Toll/IL-1R domain containing adaptor protein (TIRAP), TIR domain containing adaptor inducing interferon β (TRIF), and TRIF related adaptor molecule (TRAM) – to turn on MyD88- or TRIF-dependent pathways [3], [10], [11].

The MyD88-dependent pathway requires the recruitment of MyD88 and TIRAP, which associate with IL-1R-associated kinase (IRAK) and TNF receptor-associated factor 6 (TRAF6). These in turn activate mitogen-activated protein kinases (MAPKs), such as p38, extracellular signal-regulated kinases (ERKs), Jun N-terminal kinases (JNKs), and IκB kinase (IKK), leading to phosphorylation of the transcription factors, such as nuclear factor kappa B (NF-κB) and cAMP response element-binding protein (CREB), and then induction of genes encoding cytokines and anti-apoptotic proteins [9], [12].

In contrast, the TRIF-dependent pathway requires the recruitment of TRIF and TRAM, which bind to TNF receptor-associated factor 3 (TRAF3), leading to activation of interferon regulatory factor 3 and the expression of type I interferons (IFNs) and IFN-responsive genes [10], [11], [13]. Recent studies also indicated that the TRIF-dependent pathway mediates late-phase activation of IKK/NF-κB and MAPKs, probably through the recruitment of TRAF6 and transforming growth factor β activated kinase 1 [1].

More than 1,000 mammalian genes are induced in immune cells after stimulation with LPS, a TLR4 ligand [12], [14]. It is becoming increasingly evident that the expression of LPS-induced genes is regulated in a temporal order, and a highly integrated mechanism must ensure that the expression of these genes is ‘programmed’ after TLR4 activation [14], [15], [16]. Transcriptional control has been shown to play a crucial role in determining the kinetics of TLR4-mediated gene expression. However, NF-κB, a core transcription factor of the innate immune response, is not the only determinant of gene expression upon TLR4 engagement. Previous reports have demonstrated that, in addition to NF-κB, the expression of some LPS-induced genes in macrophages requires a second transcription factor whose activity depends on p38 [17], [18]. Several transcription factors, including CREB, ATF1, and ATF2, have been reported to be modulated by p38 kinase in TLR4-mediated immunity [19], [20]. Yet, these known p38-dependent transcription factors are not able to explain all LPS-induced genes, suggesting that a yet-to-be-identified transcription factor is involved.

Therefore, with the goal of better understanding the molecular processes underlying the temporal order of gene expression after TLR4 activation, the purpose of this study was to identify novel p38-dependent transcription factors that cooperate with NF-κB upon LPS stimulation. We used microarray analysis in combination with *in silico* analysis to identify coincident NF-κB- and p38-regulated genes. Among these genes, we demonstrated that *Tnfaip3* is regulated by both NF-κB and the p38-dependent transcription factor C/EBPβ using chromatin immunoprecipitation and functional assays.

## Materials and Methods

### Macrophage Preparation

In order to identify genes regulated by NF-κB, we conditionally knocked out *Ikkβ* (alias *Ikbkb*), encoding a catalytic enzyme in IκB kinase (IKK) complex, in *C57BL*/6 mice. *Ikbkb* ^F/F^ (*Ikkβ* ^F/F^, wild-type) and *Ikkβ* ^F/F^:*Mx1-Cre* (*Ikkβ* ^Δ^) mice have been described [21], and *C57BL*/6 mice were obtained from the Animal Center of the National Taiwan University Medical College. Mice were bred and maintained in strict accordance with the recommendations in the Guide for the Care and Use of Laboratory Animals of National Taiwan University Medical College. The protocol was approved by the Institutional Animal Care and Use Committee of National Taiwan University College of Medicine (IACUC Approval No. 20080220). Bone marrow was collected from femurs and tibia of 8–10 week-old mice and used to generate bone marrow-derived macrophages (BMDMs). Briefly, bone marrow cells were collected and cultured in high glucose Dulbecco’s modified Eagle’s medium (DMEM) (Invitrogen, Carlsbad, CA) containing 20% L929-conditioned media for 7 days with the media replaced after 4 days to stimulate differentiation into macrophages. BMDMs were then collected and cultured in DMEM with 10 ng/ml macrophage colony-stimulating factor for further experiments.

For inhibition of p38, BMDMs from C57BL/6 mice were treated with 10 µM SB202190 (Merck, Germany) for 2 h prior to use. In addition, the murine macrophage-like RAW264.7 cells (ATCC #TIB-71) were maintained in complete DMEM at 37°C in a 5% CO_2_ humidified incubator. To assess the LPS-induced macrophages, BMDMs or RAW264.7 cells were cultured with media alone or with 100 ng/ml LPS (Sigma-Aldrich, MO) for designated times before harvest.

### Microarray Experiments

Total RNA was extracted using TRIzol Reagent (Invitrogen, Carlsbad, CA) and the Qiagen RNAeasy Mini kit (Qiagen, Valencia, CA) according to the manufacturer’s instructions. RNA concentration and quality were determined using a NanoDrop ND-1000 spectrophotometer (NanoDrop Technologies, Wilmington, DE) and an Agilent 2100 Bioanalyzer (Agilent Technologies, Santa Clara, CA). Total RNA (500 ng) with A260/A280 = 1.7–2.1 and RNA integrity number >7.0 were used to synthesize the first strand cDNA via reverse transcription using an Illumina Total Pre RNA Amplification Kit (Ambion Inc., Austin, TX). Following the first strand cDNA synthesis, *in vitro* transcription was conducted using the double-stranded cDNA as a template and T7 RNA polymerase to synthesize multiple copies of biotinylated cRNA. After amplification, the cRNA was hybridized to Illumina MouseRef-8 v2 Expression BeadChips (Illumina, San Diego, CA) at 58°C for 16 h. After hybridization, the BeadChip was washed and stained with streptavidin-Cy3 dye. The intensity of the beads’ fluorescence was detected by the Illumina BeadArray Reader, and analyzed using BeadStudio v3.1 software. The microarray data of this study are MIAME compliant [22], and have been submitted to the Gene Expression Omnibus (GEO) database (accession number GSE46361).

### Microarray Data Analysis

Quantile normalization was performed using Partek Genomics Suite software (Partek, St. Louis, MO). Genes were selected as follows. Firstly, because the basal expression levels of some genes in *Ikkβ* ^Δ^ or p38-inhibited BMDMs were close to background intensity, this could result in enormous fold changes after 4 h of LPS treatment. Therefore, the basal expression levels of these genes in *Ikkβ* ^Δ^ or p38-inhibited BMDMs were replaced with those in wild-type (wt), if their expression levels before LPS treatment were not significantly different (*P*>0.05). Next, to identify LPS-responsive genes, fold changes (≥2.5x) and t-tests (*P*≤0.05) at 4 h were compared to 0 h in wt. Thirdly, to identify genes which had a suppressed LPS response in *Ikkβ* ^Δ^ or p38-inhibited cells, we selected genes whose fold changes of induction at 4 h in *Ikkβ* ^Δ^ and p38-inhibited cells were less than those in wt. Lastly, Ingenuity Pathway Analysis (IPA) (Ingenuity System, Redwood City, CA; www.ingenuity.com) was used for canonical pathway analysis. The differentially expressed genes were included in Table S1.

### Quantitative and Semi-quantitative RT-PCR

RNA was isolated using TRIzol reagent (Invitrogen, Carlsbad, CA) according to the manufacturer’s instructions. One µg of total RNA was reverse transcribed to cDNA using the High Capacity cDNA Reverse Transcription Kit (Applied Biosystems, Carlsbad, CA). cDNA aliquots equal to 50 ng of RNA were used to analyze the expression levels of mRNA. The reaction mixtures were prepared with FastStart Universal SYBR Green Master (Roche, Germany), and real-time PCR was performed with ABI 7900HT (Applied Biosystems, Carlsbad, CA). The primers used for detection of mRNAs were listed as follows: *Il-1b,* 5′-AGCCCATCCTCTGTGACTCA-3′ (forward), 5′-TGTCGTTGCTTGGTTCTCCT-3′ (reverse); *Il-6,* 5′-ATGGATGCTACCAAACTGGAT-3′ (forward), 5′-TGAAGGACTCTGGCTTTGTCT-3′ (reverse); *Serpinb2*, 5′-GTTAGAAAGTGCAAACAAGCTG-3′ (forward), 5′-GGATTTCACCTTTGGTTTGAG-3′ (reverse); *Tnfaip3,* 5′-AACCAATGGTGATGGAAACTG-3′ (forward), 5′-GTTGTCCCATTCGTCATTCC-3′ (reverse); *Zc3h12a*, 5′-CCAAGCCTTCCACTCTAGAAC-3′ (forward), 5′-GGCACAAACACGGTAATATCTG-3′ (reverse); *Cypa* (*Cyclophilin A*), 5′-ATGGTCAACCCCACCGTGT-3′ (forward), 5′-TTTCTGCTGTCTTTGGAACTTTGTC-3′ (reverse). Relative quantities of mRNAs were normalized to 18S rRNA or Cyclophilin A, and the fold inductions were determined. For semiquantitative RT-PCR, equal amounts of cDNA were subjected to regular PCR with variable cycles, and PCR products were then analyzed by electrophoresis on a 1.5% agarose gel after staining with ethidium bromide.

### Prediction of Transcription Factor Binding Sites

oPOSSUM (http://www.cisreg.ca/oPOSSUM/) [23] was used to predict the transcription factor binding sites in *Tnfaip3*. The JASPAR CORE vertebrate database was selected for transcription factor binding site matrices, and the selection parameters were set as follows: top 10% for conserved regions, 80% matrix match, and 2000/0 for upstream/downstream sequence length.

### shRNA Mediated Gene Silencing against *Cebpb*

HEK293T packaging cells (ATCC # CRL-11268) were cultured in high-glucose DMEM supplemented with 10% FBS. Transfection of HEK293T cells was conducted using Turbofect (Thermo Scientific) according to the manufacturer’s instructions. The specific lentiviral shRNA constructs against *Cebpb* were obtained from the National RNAi Core Facility in Taiwan. Their target sequences are: *shCebpb* #1, CACCCTGCGGAACTTGTTCAA; *shCebpb* #2, CAAGGCCAAGATGCGCAACCT. Lentivirus was packaged into HEK293T cells following the guidelines of National RNAi Core Facility (http://rnai.genmed.sinica.edu.tw/protocols), and the culture supernatants containing the lentivirus were collected at 48 and 72 h post-transfection. RAW264.7 cells were infected with lentiviruses in the presence of 8 µg/ml polybrene (Sigma) overnight and cultured in fresh medium for another 24 h. The infected cells were then selected in medium containing 0.4 µg/ml puromycin until the uninfected cells were completely killed.

### Chromatin Immunoprecipitation

Chromatin immunoprecipitation (ChIP) was performed using a Magna ChIP kit (Millipore, MA) according to the manufacturer’s instructions. In brief, RAW264.7 cells were fixed with formaldehyde for 10 min to cross-link DNA and proteins and washed with cold PBS. Cells were then lysed using cell and nuclear lysis buffer and sheared on ice with a sonicator to generate DNA fragments from 200 to 1,000 bp. The resulting suspension was used for immunoprecipitation by incubating overnight at 4°C with protein G magnetic beads and the following antibodies: rabbit anti-NF-κB p65 (sc-372, Santa Cruz), rabbit anti-C/EBPβ (sc-150, Santa Cruz), and normal rabbit IgG (sc-2027, Santa Cruz). Approximately 1% of the suspension was removed before immunoprecipitation to determine the input quantity of DNA. The precipitated DNA-protein complexes were washed repeatedly with wash buffer, and cross-links were reversed by incubating with elution buffer and proteinase K at 65°C for 2 h. DNA was purified by purification columns provided in the kit and analyzed by semi-quantitative PCR. Primers specific to the predicted binding sites in promoter regions of *Tnfaip3* were designed to amplify a fragment spanning positions −89 to −410∶5′-CCCGGAGAAACTCCTAGGTC-3′ (forward); 5′-GCCGCTTTTTCTGTCAATTC-3′ (reverse).

### Immunoblotting

Cells were lysed and cell extracts were collected. Protein concentrations were determined by the Bradford assay (Bio-Rad). Cell lysates were then resolved by SDS-PAGE and transferred to polyvinylidene difluoride (PVDF) membranes (Millipore). The membranes were then incubated with the indicated primary antibody followed by an HRP-conjugated secondary antibody. The antibodies are: rabbit anti-C/EBPδ (sc-636, Santa Cruz); p38 (sc-728, Santa Cruz); IKKβ (05–535, Upstate); A20 (IMG-161, Imgenex); GAPDH (#5174, Cell Signaling); phospho-p38 (#9211, Cell Signaling); actin (A4700, Sigma); and HRP-conjugated anti-mouse and anti-rabbit secondary antibodies (115-035-003 and 111-035-003, Jackson ImmunoResearch). The immunoreactive bands were detected using the Western Lighting® Plus-ECL (PerkinElmer).

### Statistical Analysis

All statistical analyses were performed with SPSS 13.0. Data are presented as means ± standard deviation (SD) from at least two separate experiments. Statistical significance was determined by Student’s t test. Unless otherwise indicated, a *P* value less than 0.05 was considered significant.

## Results

### Inhibition of NF-κB and p38 Signaling Pathways in LPS-induced Bone Marrow Derived Macrophages

**Figure 1:**
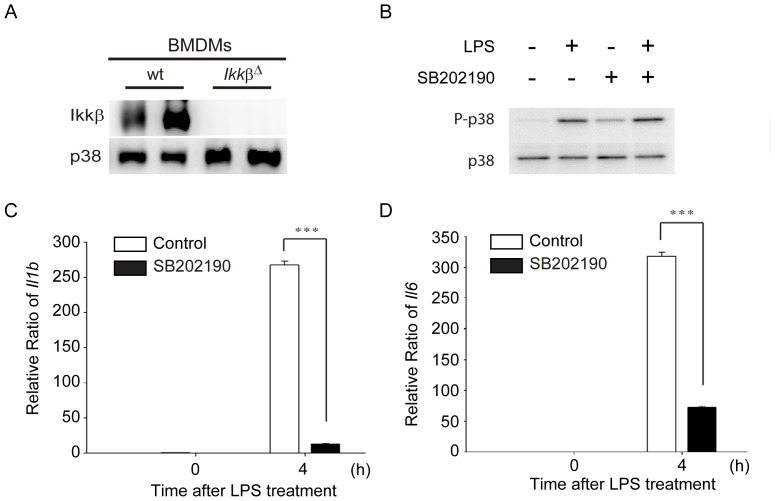
Depletion of IKKβ expression and inhibition of p38 signaling pathway in *Ikkβ* ^Δ^ and SB202190-treated bone marrow-derived macrophages (BMDMs). (A) Immunoblotting of IKKβ and p38 from BMDMs isolated from wild-type (wt; *Ikkβ* ^F/F^) and *Ikkβ* ^Δ^ mice. (B) Immunoblotting of p38 and phosphorylated p38 from wt BMDMs treated with LPS (100 ng/mL) in the absence or presence of SB202190 (10 µM) for 2 h. (C & D) mRNA expression levels of IL-1β and IL-6 were inhibited in SB202190-treated BMDMs after LPS treatment. The expression levels of *Il1b* (C) and *Il6* (D) were determined from wt BMDMs treated with LPS (100 ng/mL) for 4 h in the absence or presence of SB202190 (10 µM) for 2 h using real-time RT-PCR. Data represent the mean ± SEM for three independent experiments. ***, *P*<0.005.

Since NF-κB is retained in the cytoplasm through association with inhibitor κB (IκB) and degradation of IκB depends mainly on IKKβ [24], BMDMs derived from *Ikkβ* ^Δ^ mice were used to identify genes regulated by NF-κB. To identify the differentially expressed genes in wt and IKKβ-deficient BMDMs, BMDMs generated from wt (*Ikkβ* ^F/F^; *Ikkβ* flanked with *LoxP* sites) and *Ikkβ* ^Δ^ mice were cultured and treated with 100 ng/ml LPS for 2, 4, and 8 hours or with medium alone as a control. RNAs extracted from these BMDMs were analyzed using an Illumina MouseRef-8 v2 Expression BeadChip, which provides 25,697 probes and targets over 19,100 unique genes. To assess the depletion of *Ikkβ* (alias *Ikbkb*) in these *Ikkβ* ^Δ^ BMDMs, we examined the mRNA expression levels of *Ikkβ* in wt and *Ikkβ* ^Δ^ BMDMs. As shown in Fig. 1A, western blotting showed that protein amounts of IKKβ were hardly detected in *Ikkβ* ^Δ^ BMDMs as compared to wt BMDMs, indicating the success of *Ikkβ* depletion. Furthermore, a pilot study in microarray analysis showed that most genes were induced by LPS at 4 h (data not shown). We therefore focused on the 4-hour time point of LPS treatment in subsequent experiments.

To explore which genes respond to LPS via the p38 signaling pathway, BMDMs of C57BL/6 mice were preincubated with 10 µM p38 inhibitor SB202190 for 2 h, followed by 4 h of LPS treatment. SB202190 treatment did not suppress TLR4-activated proteins upstream of p38 MAPK activity, demonstrated by phosphorylation of p38 at Thr180/Tyr182 (Fig. 1B). To confirm that p38 kinase activity was inhibited by SB202190, mRNA expression levels of IL-1β (Interleukin-1β, encoded by *Il1b*) and IL-6 (Interleukin 6, encoded by *Il6*), cytokines produced by the activation of the p38 pathway [25], were analyzed by quantitative RT-PCR. The results revealed that both *Il1b* (Fig. 1C) and *Il6* (Fig. 1D) mRNAs were significantly (*P*<0.005) suppressed in BMDMs pretreated with SB202190.

### Identification of p38-dependent NF-κB Target Genes in Activated Macrophages

**Figure 2:**
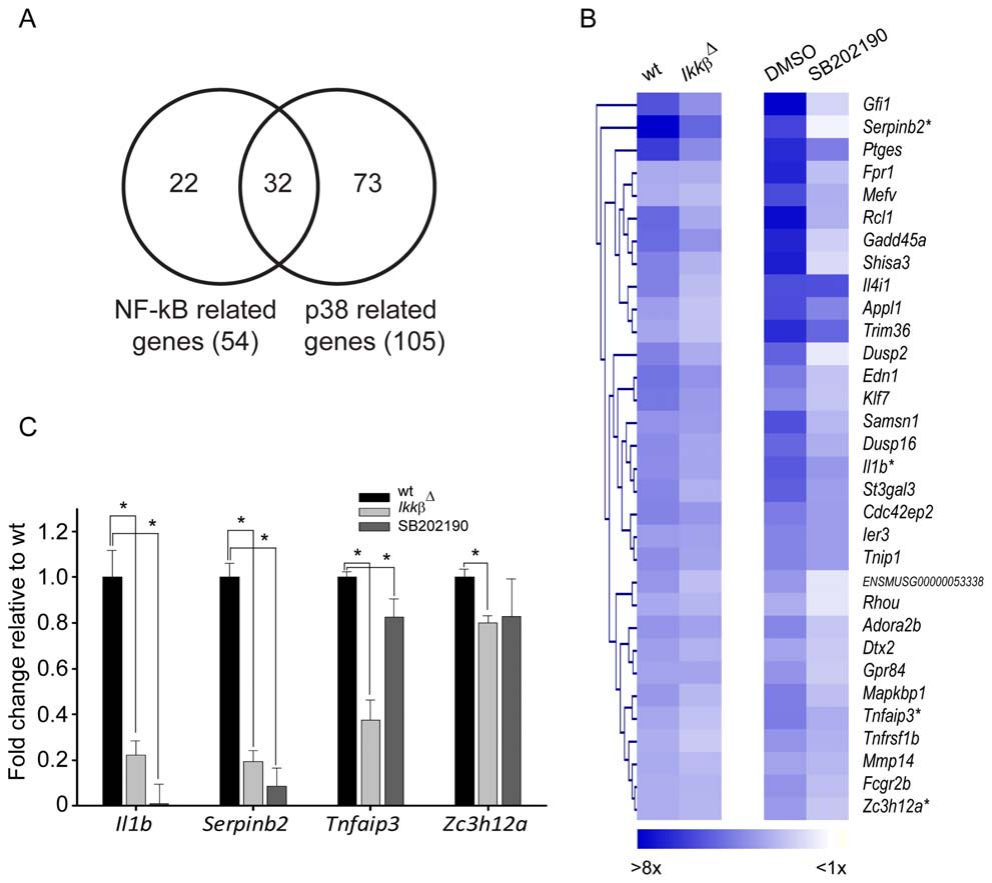
Identification of LPS-induced genes that were regulated by both NF-κB and p38. (A) Venn diagram of NF-κB and p38-dependent genes. NF-κB-related genes were identified from genes that were down-regulated in *Ikkβ* ^Δ^ BMDMs as compared with wt BMDMs after LPS treatment, and p38-related genes were selected by comparing SB202190- (p38-inhibitor) with dimethyl sulfoxide (DMSO)-treated BMDMs after LPS treatment. Thirty-two LPS-induced genes were regulated by both NF-κB and p38-downstream transcription factors. (B) Hierarchical clustering of average fold change for the NF-κB and p38-dependent genes. Each column represents the average fold change 4 h after LPS treatment compared to 0 h. *: genes chosen for PCR validation. (C) Relative fold changes of *Il1b, Serpinb2, Tnfaip3*, and *Zc3h12a* mRNA from BMDMs from wt and *Ikkβ* ^Δ^ cells stimulated with LPS (100 ng/mL) for 4 h in the presence or absence of SB202190 (10 µM) were measured by quantitative RT-PCR. The fold change of 4 h vs. 0 h was normalized to wt BMDMs. The internal control was Cyclophilin A mRNA (*Cypa*). Data represent the mean ± SD for at least two independent experiments. *, *P*<0.05.

To investigate genes that were regulated by both NF-κB and p38-downstream transcription factors in activated macrophages, we first used microarrays to identify genes induced by LPS in a NF-κB- and p38-dependent manner. All microarray data were first subjected to quantile normalization. In order to identify NF-κB regulated genes, we sought genes that were down-regulated in *Ikkβ* ^Δ^ BMDMs as compared to wt BMDMs in response to LPS. Similarly, genes that were regulated by p38-dependent transcription factors were identified by comparing SB202190-treated BMDMs with untreated BMDMs. Afterward, genes identified in both comparisons were chosen for further analysis. The selection criteria of gene candidates were described in *Materials and Methods*. Briefly, since the basal expression of some genes in *Ikkβ* ^Δ^ or SB202190-treated BMDMs was close to background intensity levels, we used wt as the basal expression levels, if no significant differences were found among wt, *Ikkβ* ^Δ^,or p38-inhibited BMDMs before LPS treatment. The NF-κB and p38-dependent genes were genes that were significantly (*P*≤0.05) up-regulated by at least 2.5-fold after induction by LPS in wt, and suppressed in *Ikkβ* ^Δ^ and p38-inhibited BMDMs. As shown in Fig. 2A, 54 genes were identified as NF-κB regulated genes in the wt vs. *Ikkβ* ^Δ^ comparison, and 105 genes were selected as p38-dependent genes in the wt vs. p38-inhibited comparison. Among them, 32 genes in common were both NF-κB- and p38-dependent genes. As shown in Fig. 2B, the average fold changes of these 32 genes were higher at 4 h after LPS in wt or DMSO as compared to those in *Ikkβ* ^Δ^ or SB202190-treated cells. The expression values of selected genes, including *Il1b, Serpinb2, Tnfaip3,* and *Zc3h12a*, were validated by real-time RT-PCR. As shown in Fig. 2C, these genes were significantly down-regulated in *Ikkβ* ^Δ^ and in the presence of p38 inhibitor SB202190.

### Functional Analysis of the NF-κB and p38-dependent Genes

To investigate the canonical pathways of these 32 genes, Ingenuity Pathway Analysis (IPA) was used. Not surprisingly, the results revealed that the top 10 canonical pathways were mostly related to the inflammatory response, such as NF-κB signaling, granulocyte adhesion and diapedesis, TNFR2 signaling, dendritic cell maturation, etc. (Table 1).

**Table 1:** Top 10 enriched canonical pathways of the NF-κB and p38-dependent genes.

Rank	Ingenuity Canonical Pathways	−log(*P*-value)^a^	Genes
1	NF-κB signaling	3.64	*Tnip1,Tnfaip3,Il1b,Tnfrsf1b*
2	Granulocyte adhesion and diapedesis	3.62	*Mmp14,Il1b,Tnfrsf1b,Fpr1*
3	TNFR2 signaling	2.90	*Tnfaip3,Tnfrsf1b*
4	Hepatic fibrosis/hepatic stellate cell activation	2.67	*Edn1,Il1b,Tnfrsf1b*
5	Dendritic cell maturation	2.40	*Il1b,Fcgr2b,Tnfrsf1b*
6	TREM1 signaling	2.32	*Il1b,Fcgr2b*
7	IL-10 signaling	2.14	*Il1b,Fcgr2b*
8	Role of osteoblasts, osteoclasts and chondrocytes in rheumatoid arthritis	2.12	*Mmp14,Il1b,Tnfrsf1b*
9	Colorectal cancer metastasis signaling	2.03	*Appl1,Mmp14,Rhou*
10	PPAR signaling	1.90	*Il1b,Tnfrsf1b*

^a^The significance level of each canonical pathway was determined by in Ingenuity Pathway Analysis.

### LPS-induced Recruitment of p65 and C/EBPβ to the *Tnfaip3* Promoter and Upregulation of A20 Expression

Since co-expressed genes in the same functional pathway are usually subject to similar transcriptional regulation, there should be an over-representation of NF-κB and p38-related transcription factor binding sites in the promoters of a set of co-expressed genes [26], [27]. Therefore, to validate that these genes were regulated by both NF-κB and p38-related transcription factors, we used the oPOSSUM website to search for binding sites of NF-κB and p38-related transcription factors in the promoters of the NF-κB and p38-dependent genes. The sequences from −2,000 to +0 bp in the promoter regions of these genes were searched using the JASPAR database, and the prediction parameters were set as described in *Materials and Methods*.

As shown in Table 2, NF-κB and its family members, such as RELA (p65), REL, NFKB1, and NF-κB (p50) were identified at the top of the list. The next two transcription factors were DDIT3-C/EBPα dimers and C/EBPα. C/EBPα belongs to the C/EBP family and shares the same binding motif with other members of the family, such as C/EBPβ, C/EBPγ and C/EBPδ. Since it has been reported that MAPKs modulate C/EBPβ activity [28], we postulated that the NF-κB and p38-dependent genes were co-regulated by NF-κB and p38-regulated C/EBPβ. Indeed, 10 genes – *Dusp16*, *Edn1, Gadd45a, Gfi1, Klf7, Mapkbp1, Rcl1, Tnfaip3, Tnip1,* and *Zc3h12a* – had both NF-κB and C/EBP binding sites in their promoter regions.

**Table 2:** Prediction of transcription factor binding sites of NF-κB and p38-dependent genes using opossum.

TF§	TF Class	Background TFBS rate†	Target TFBS rate		Z-score		
RELA (p65)	REL	0.0043		0.0152		17.64	
REL	REL	0.0094		0.0241		16.04	
NFKB1	REL	0.0028		0.0108		15.98	
NF-κB (p50)	REL	0.0061		0.0152		12.27	
DDIT3-C/EBPα	bZIP	0.0036		0.0086		8.55	
C/EBPα	bZIP	0.0114		0.0193		7.80	
HLF#	bZIP	0.0048		0.0096		7.27	
SRF#	MADS	0.0005		0.0021		7.19	
USF1#	bHLH-ZIP	0.0083		0.0144		6.95	
SOX9	High Mobility Group	0.0115		0.0185		6.87	

§ TF: transcription factor.

† Background TFBS rate: rate of transcription factor binding site in genome.

# TF Abbreviation: HLF: hepatic leukemia factor; SRF: serum response factor; USF1: Upstream stimulatory factor 1.

**Figure 3:**
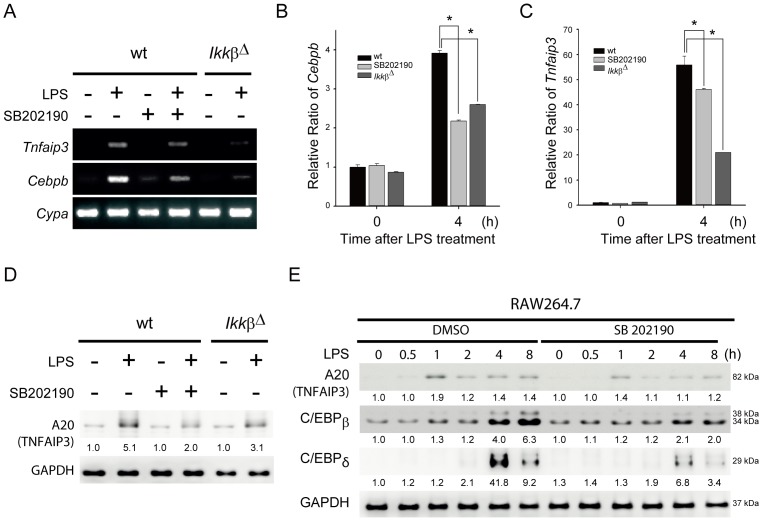
C/EBPβ and A20 (TNFAIP3) were suppressed in *Ikkβ* ^Δ^ and p38-inhibited macrophages. (A–C) Expression levels of *Tnfaip3* and *Cebpb* mRNA were decreased in *Ikkβ* ^Δ^ and p38-inhibited BMDMs in response to LPS. BMDMs from wt and *Ikkβ* ^Δ^ cells treated with or without SB202190 (10 µM) were stimulated with LPS (100 ng/mL) for 4 h. Total RNAs were isolated and analyzed by semi-quantitative RT-PCR (A) or quantitative real-time RT-PCR for expression of *Cebpb* (B) and *Tnfaip3* (C) mRNAs. Results were normalized to Cyclophilin A (*Cypa*) and are presented relative to expression in wt BMDMs. *P<0.05. (D) A20 (TNFAIP3) protein levels were decreased in p38-inhibited (SB202190) and *Ikkβ* ^Δ^ BMDMs after LPS treatment. (E) Temporal profiles of A20, C/EBPβ, and C/EBPδ in p38-inhibited RAW264.7 cells after LPS treatment for the indicated duration. Cell lysates were prepared and analyzed by immunoblotting with the indicated antibodies.

To investigate the binding activities of NF-κB and C/EBPβ in the promoters of these genes, we chose *Tnfaip3*, which encodes the ubiquitin-modifying enzyme A20, as a target gene for further experiments based on the number of binding sites and their proximity to the transcription start site. First, we examined whether C/EBPβ and A20 were suppressed in *Ikkβ* ^Δ^ and p38-inhibited cells by RT-PCR (Fig. 3A, B, & C) and immunoblotting (Fig. 3D). Expression of *Cebpb* and *Tnfaip3* were significantly inhibited both in p38-inhibited BMDMs and in *Ikkβ* ^Δ^ BMDMs 4 hours after LPS treatment (Fig. 3A, B, & C). Also, protein amounts of A20 (TNFAIP3) decreased both in p38-inhibited BMDMs and in *Ikkβ* ^Δ^ BMDMs (Fig. 3D). Furthermore, using the murine macrophage cell line RAW264.7, both A20 and C/EBPβ showed similarly reduced expression patterns starting from 1 hour after LPS treatment in p38-inhibited cells (Fig. 3E). Consistent with previous reports [29], [30], C/EBPδ, another C/EBP family transcription factor whose induction is also dependent on p38 MAPK, was induced at 4 h after LPS treatment, suggesting that C/EBPδ is unlikely to be responsible for LPS-triggered A20 expression.

**Figure 4:**
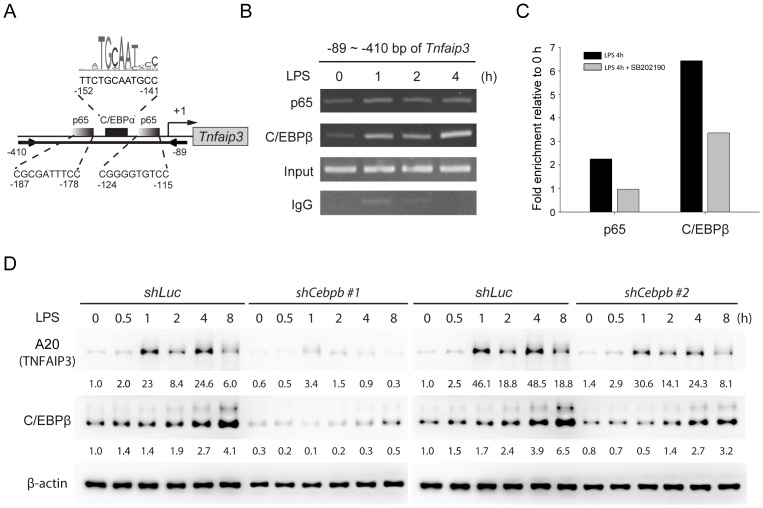
NF-κB and C/EBPβ are required for induction of *Tnfaip3* in LPS-activated macrophages. (A) Schematic map of predicted NF-κB p65 and C/EBPβ DNA binding sites in the promoter of *Tnfaip3*. oPOSSUM (http://www.cisreg.ca/oPOSSUM/) was used as the prediction tool. The JASPAR CORE vertebrate database was selected for transcription factor binding site matrices. Two arrows depict the PCR primers. (B) p65 and C/EBPβ bind to the promoter of *Tnfaip3* after LPS stimulation. RAW264.7 cells were treated with LPS (100 ng/ml) for the indicated times. Chromatin was immunoprecipitated with anti-p65 and anti-C/EBPβ antibodies. Rabbit IgG was a negative control. Precipitated DNA or 1% of the chromatin input was amplified with primers for the *Tnfaip3* promoter (−89 ∼ −410). The PCR products were loaded and separated on a 2% agarose gel. One of two independent experiments is shown. (C) LPS-induced association of p65 and C/EBPβ with *Tnfaip3* was reduced in the presence of p38 inhibition. Chromatin isolated from RAW264.7 cells treated with LPS (100 ng/ml) for 4 h in the absence or presence of SB202190 (10 µM) were subjected to ChIP assay as described above. The relative quantity of promoter enriched by ChIP was quantified by real-time PCR and expressed as the fold enrichment of untreated control samples after normalization to rabbit IgG. Data represent the means of two independent experiments. (D) LPS-induced expression of A20 (TNFAIP3) was decreased in C/EBPβ-depleted RAW264.7 cells. Cells were infected with lentiviruses encoding shRNA against luciferase (*shLuc*) or C/EBPβ (*shCebpb*), and treated with LPS (100 ng/ml) for the indicated times. Cell lysates were collected and analyzed by immunoblotting using the indicated antibodies.

Next, chromatin immunoprecipitation (ChIP) assays using anti-p65 or anti-C/EBPβ antibodies were performed in RAW264.7 cells stimulated with LPS for 0, 1, 2 and 4 hours. Subsequent PCR was done to amplify a fragment (−89 ∼ −410 bp) of the *Tnfaip3* promoter containing p65 and C/EBPβ binding sites (Fig. 4A). Recruitment of p65 and C/EBPβ to the *Tnfaip3* promoter was confirmed, with slightly increased binding of p65 and obviously increased binding of C/EBPβ upon exposure to LPS (Fig. 4B). Real-time PCR analysis of ChIP showed that p65 and C/EBPβ associated with the *Tnfaip3* promoter after LPS treatment in control RAW264.7 cells, and that the association was reduced upon p38 inhibition (Fig. 4C). To further confirm that C/EBPβ is involved in TLR4-activated A20 expression, we depleted C/EBPβ expression in RAW264.7 cells by lentivirus-mediated short hairpin RNA (shRNA). Stimulation of cells expressing control shRNA (*shLuc*) with LPS induced A20 production, whereas C/EBPβ-depleted RAW264.7 cells showed decreased levels of A20 in response to LPS (Fig. 4D). Together these data indicate that NF-κB p65 and C/EBPβ were mediators of LPS-induced *Tnfaip3* expression in macrophages.

## Discussion

The expression levels of LPS-induced genes in macrophages are strictly regulated by NF-κB and other transcription factors whose activities depend on p38 MAP kinase [17], [18]. However, these transcription factors remain to be identified. In this study, microarrays were used to identify genes regulated by both NF-κB and p38. *In silico* analysis of transcription factor binding sites was used to predict the potential synergistic transcription factors from the co-regulated genes. Among these genes, we found that NF-κB and C/EBPβ, a p38-downstream transcription factor, co-regulate *Tnfaip3* in response to LPS treatment in macrophages.

Although some studies have investigated global expression profiling using cDNA microarrays to understand the molecular processes of gene expression after LPS stimulation [12], [14], [15], [16], [31], they either discussed general patterns of expression profiling or were narrowly focused on a particular physiological function. For example, Park JM et al. found that the TLR4-p38 MAPK-CREB axis is responsible for PAI-2 induction by searching p38-dependent transcription factors in LPS-induced apoptotic gene promoters [12]. In contrast, we used a systematic approach to lead the discovery of LPS-responsive genes regulated by NF-κB and a p38-downstream transcription factor C/EBPβ. By comparing the mRNA expression levels in wt cells with those in *Ikkβ* ^Δ^ and in p38-inhibited cells, 32 LPS-induced genes subject to NF-κB- and p38-mediated regulation were identified. Furthermore, *in silico* analysis predicted that 10 genes (*Dusp16*, *Edn1, Gadd45a, Gfi1, Klf7, Mapkbp1, Rcl1, Tnfaip3, Tnip1,* and *Zc3h12a*) contained NF-κB and C/EBP binding sites in their promoters. These genes are very likely subject to NF-κB and C/EBPβ regulation in response to LPS stimulation. Yet, due to the limitations of bioinformatics, this hypothesis still needs to be experimentally validated. In addition, although using primary BMDMs for experimentation has always been a high priority, the resources were limited. Therefore, the murine macrophage-like RAW264.7 cells were used in some experiments of this study.

It has been estimated that nearly 100 transcription factors are induced by LPS to precisely regulate the high complexity of TLR4-induced responses [29]. In our search to identify novel transcription factors, promoters from co-expressed genes were searched for over-represented sequence motifs. The basic assumption of this search was that genes showing similar expression patterns should be regulated by the same transcription factors, and, therefore, the promoters of co-expressed genes should contain similar sequence elements, corresponding to binding sites for the common regulators. In addition to NF-κB and its family members, C/EBP binding sites were predicted in gene promoters and identified as potential p38-activated transcriptional regulation sites. In order to validate these genes that were regulated both by NF-κB and C/EBPβ, we chose *Tnfaip3* for further experiments based on the number of binding sites and their proximity to the transcription start site. Previously, Litvak et al. also identified a C/EBPβ binding motif in the promoter of *Tnfaip3* [29].

C/EBP is a family of transcription factors that share a highly conserved dimerization domain required for DNA binding and have been shown to be dependent on interaction with other transcription factors, including NF-κB, Sp1, and Fos/Jun [32], [33]. For example, LPS can enhance the gene expression of *FLAP* via both NF-κB and C/EBP in phagocytes [34]. Also, the C/EBP families of transcription factors have been shown to participate in regulating proinflammatory cytokine expression upon TLR activation [28], [30]. Likewise, the C/EBP binding motif can be found in the promoters of many LPS-induced genes. It has been shown that LPS/TLR4-activated C/EBPβ is dependent on the MyD88/IRAK4 pathway [30]. Nevertheless, the mechanism of LPS-stimulated C/EBP remains incompletely understood [35].

P38 MAPK has been proven to be required for full transcriptional activation of several TLR4-activated genes in dendritic cells [36] and in macrophages [12]. Previous studies have showed *Helicobacter pylori* LPS is able to activate C/EBPβ through PI3K/Akt/p38 MAPK signaling to drive pro-IL-1β transcription [37]. However, since *H. pylori* LPS does not trigger the typical TLR4 pathway [38], it remains to be further elucidated whether the PI3K/Akt/p38 MAPK axis is involved TLR4-induced C/EBPβ expression. In this study, we demonstrated a novel mechanism of TLR4-induced C/EBPβ activity through p38 MAPK, possibly in a MyD88-dependent manner, which subsequently regulates A20 transcription in conjunction with NF-κB.

A growing body of research has reported that C/EBPβ activity can be mediated through transcriptional, post-transcriptional, translational, and post-translational mechanisms, including increased C/EBPβ protein levels by induction of C/EBPβ transcription, regulation of nuclear localization, alternative translation initiation, and phosphorylation by various kinases [28]. Our data showed that LPS up-regulated the levels of C/EBP mRNA and protein, and p38 inhibition suppressed this effect, leading to decreased A20 expression. In line with our data, previous studies revealed that LPS was able to induce C/EBPβ transcription, but not alternative translation initiation, nuclear translocation, or post-translational modifications in J774 macrophage cells [28]. These results together suggest that upon TLR4 activation p38 modulates C/EBPβ activity by increasing its transcription and protein levels.

The *Cebpb gene* encodes a single transcript which is translated into three isoforms due to alternative use of different AUG initiation codons; 38 kDa and 34 kDa, the liver-enriched transcriptional activating proteins (LAPs), and 20 kDa, the liver-enriched transcription inhibitory protein (LIP), which acts as a negative transcriptional repressor [39]. Our results showed that both 38 and 34 kDa LAPs were induced by LPS and that the 34 kDa LAP is the predominant form. Recent studies reported that the 34 kDa C/EBPβ form is responsible for its transcriptional activation in LPS-activated macrophages [39]. Thus, it is likely that the 34 kDa C/EBPβ is the key factor regulating LPS-triggered A20 expression. However, we cannot rule out the involvement of the 38 kDa C/EBPβ form in induction of A20.

In addition, C/EBPδ was previously shown to be activated by LPS and subsequently regulated several TLR4-mediated gene expressions [29]. Our results also showed that LPS indeed induced C/EBPδ at 4 h after LPS treatment, whereas expression of C/EBPβ and A20 preceded C/EBPδ, indicating that C/EBPβ, but not C/EBPδ, is likely the key isoform responsible for LPS-induced A20 expression. This result was consistent with a recent microarray analysis that A20 was not identified as a C/EBPδ-mediated gene in LPS-treated macrophages [29].

TNFα-induced protein 3 (TNFAIP3, also known as A20, a ubiquitin-modifying enzyme) is a cytoplasmic zinc finger protein that has been characterized as a dual inhibitor of NF-κB activation and cell death [40]. A20 knockout mice were more susceptible to TNFα-induced inflammation and demonstrated premature death due to severe septic shock [41]. Mechanistically, A20-deficent fibroblasts were not able to terminate TNF-induced NF-κB activity, leading to TNF-mediated apoptosis [41]. In addition to its effects on the TNF-induced inflammatory response, A20 was found to be up-regulated in mouse BMDMs after stimulation with LPS and was required for termination of TLR responses through its de-ubiquitination activity on TRAF6 [42]. Thus, induction of A20 upon TLR4 activation functions in the negative feedback regulation of NF-κB and IRF3 activation [39]–[41]. However, the molecular mechanism of TLR4-induced A20 expression is not well understood. Our present studies should be able to provide some insight into part of this mechanism.

**Figure 5:**
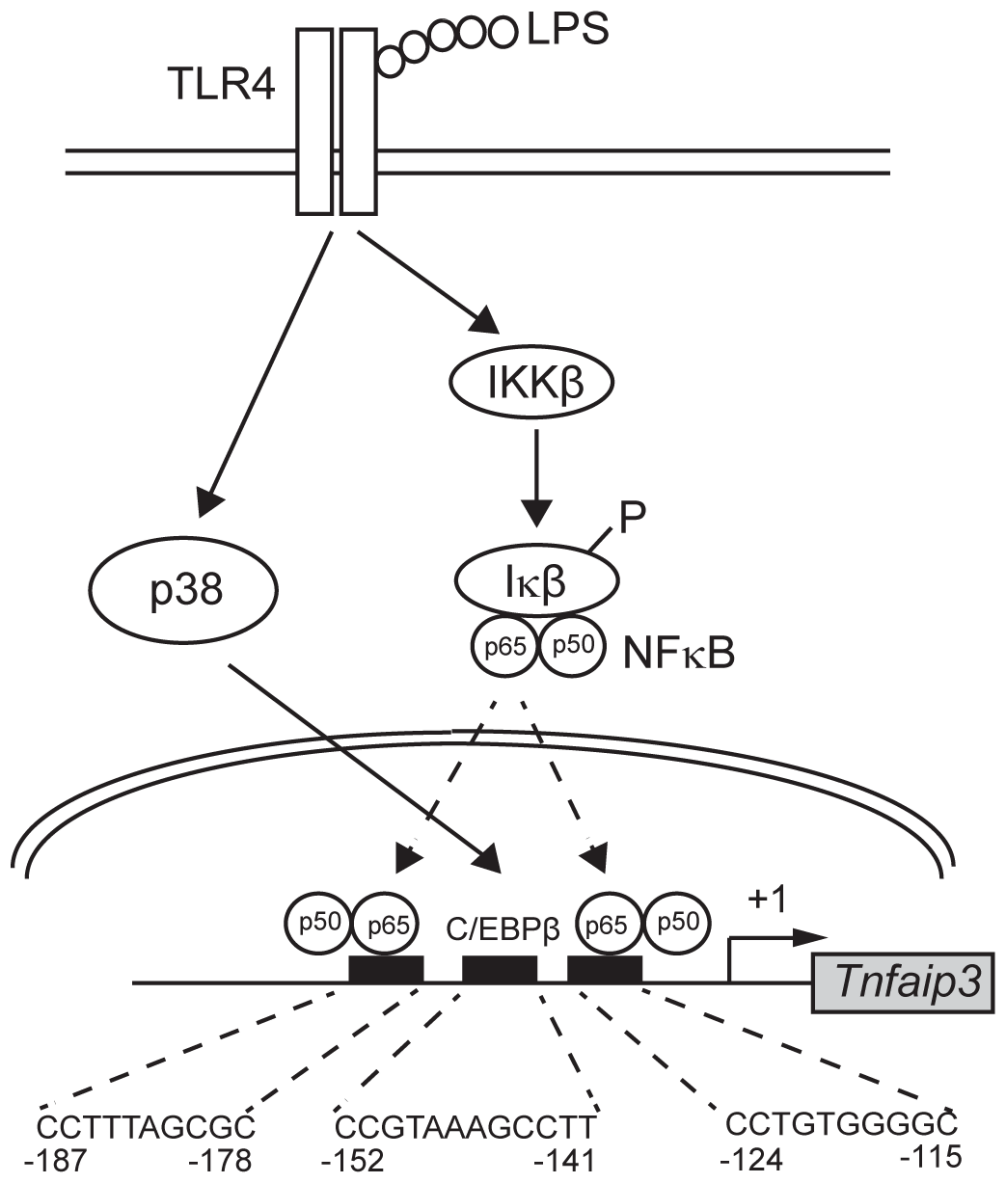
Proposed model of NF-κB and p38 via C/EBPβ regulating the transcription of *Tnfaip3* in LPS-induced response. TLR4 engagement leads to activation of p38 MAPK and IKK/NF-κB. p38 MPAK subsequently through a yet-to-be-determined mechanism upregulates C/EBPβ, which induces A20 (TNFAIP3) transcription in conjunction with NF-κB.

From the results of semi-quantitative PCR analysis of the ChIP assay, C/EBPβ had the highest binding activity on the *Tnfaip3* gene promoter after 4 h of LPS treatment, whereas p65, a subunit of NFκB transcription complex, only showed slightly increased binding after LPS induction. In addition, p38 inhibition in RAW264.7 cells decreased the binding activity of C/EBPβ and p65 at 4 h after LPS treatment. To explain why the binding of p65 was also suppressed in the presence of p38 inhibition, we presumed that p65 might interact with C/EBPβ. We have proposed a working model based on these results (Fig. 5) and suggest that *Tnfaip3* might be expressed through transcriptional regulation of both p65 and the p38-downstream transcription factor C/EBPβ in response to LPS.

## Supporting Information

Table S1 **Supplementary materials.** The Excel file contains gene list and relative fold changes of P38 & NF-κB dependent genes, NF-κB related probes, and P38 related probes in three spreadsheets.
(XLS)
